# Diagnosis and treatment of appendiceal mucocele: a rare cause of intestinal blockage

**DOI:** 10.1093/jscr/rjaf296

**Published:** 2025-05-12

**Authors:** Emmanuel García Romero, Rubén Daniel Pérez López, Yunuen Ailyn Morales Tercero

**Affiliations:** Department of General Surgery, Universidad Nacional Autónoma de México, Mexico City 04360, Mexico; Department of General Surgery, Universidad Nacional Autónoma de México, Mexico City 04360, Mexico; Department of General Surgery, Universidad Nacional Autónoma de México, Mexico City 04360, Mexico

**Keywords:** inguinal hernia, bladder herniation, laparoscopic surgery, transabdominal preperitoneal (TAPP) approach, diagnostic imaging

## Abstract

Inguinal hernias are common in surgical practice, with a small percentage involving bladder herniation. These inguinoscrotal bladder hernias, though rare, present significant diagnostic and treatment challenges. This case report details the diagnosis, treatment, and postoperative management of a 63-year-old male with benign prostatic hyperplasia, presenting with an inguinal hernia involving the bladder. Diagnosis was confirmed with physical examination and computed tomography scans, showing bladder herniation into the inguinal canal. Surgery involved laparoscopic inguinal hernia repair using a transabdominal preperitoneal approach. The surgery was successful with no complications and the patient was discharged 48 hours later. A three-month follow-up showed no recurrence or urinary complications. This case emphasizes the importance of considering inguinoscrotal bladder hernias in patients with inguinal bulges and urinary symptoms. Early diagnosis, supported by imaging and awareness, followed by laparoscopic repair, is essential for favorable outcomes in these rare cases.

## Introduction

The appendiceal mucocele, first described by Rokitansky in 1842, occurs in 0.2% to 0.7% of dissected appendix specimens. This condition is characterized by the obstructive, distended appearance of the appendix due to mucous accumulation, which can be benign or malignant [1]. In 2012, the Peritoneal Surface Oncology Group International proposed a consensus classification [2]: non-neoplastic mucoceles, retention cysts, and inflammatory or obstructive mucoceles without mucosal hyperplasia or neoplasia; and neoplastic forms, including serrated polyps with or without dysplasia, low- and high-grade mucinous neoplasms, and mucinous adenocarcinoma. This condition typically presents in patients over 50, with symptoms that are often late and vague, such as lower abdominal pain or a palpable mass, which can mimic appendicitis or a tubo-ovarian mass in women [1]. In a Spanish case series, 45% of cases presented with acute appendicitis, 35% with a mass in the right iliac fossa, and 10% were incidental findings [3]. Intestinal obstruction due to appendiceal masses is rare.

## Case report

A 74-year-old female with no significant personal medical history presented to the emergency department reporting progressive onset of diffuse abdominal pain of moderate to severe colicky spasm type that began one day prior. Associated symptoms included abdominal distension, oral intolerance with nausea and vomiting on multiple occasions, hyporexia, and constipation.

Vital signs were within normal limits but showed a trend towards hemodynamic instability, which progressed without response to fluids, prompting the initiation of norepinephrine at a dose responsive to the situation. Physical examination revealed a distended, tense abdomen with metallic sounding bowel sounds, tympanic on percussion, painful on palpation, with a positive rebound and signs of peritoneal irritation.

Laboratory tests showed no abnormalities: white blood cells at 4.7 × 10^3^/μl, neutrophils at 73%, hemoglobin at 14.4 g/dl, hematocrit at 43.1%, platelets at 161 000 × 10^3^/μl, creatinine at 0.5 mg/dl, glucose at 113 mg/dl, urea at 34.1 mg/dl, sodium at 136 mmol/L, potassium at 4.1 mmol/L, and calcium at 9.9 mg/dl.

**Figure 1:**
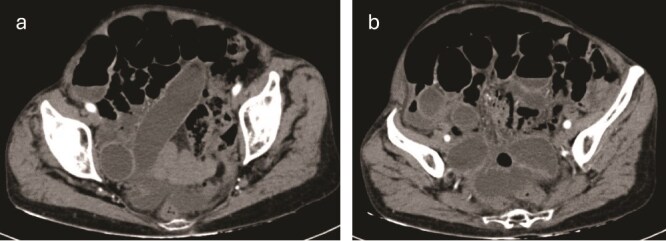
(a, b) Contrast-enhanced CT of the abdomen revealed loops of small intestine with dilation up to 31 mm and the presence of a whirl sign at the level of the distal ileum associated with a transition zone.

Contrast-enhanced computed tomography (CT) of the abdomen revealed loops of small intestine with dilation up to 31 mm and the presence of a whirl sign at the level of the distal ileum associated with a transition zone. Sigmoid diverticulosis and free fluid in the peritoneal cavity were noted, distributed in the anterior subhepatic space, both paracolic gutters, and scantily among intestinal loops. Imaging suggested considerations of a midgut volvulus versus adhesions in the topography of the distal ileum (Fig. 1 a and b).

**Figure 2:**
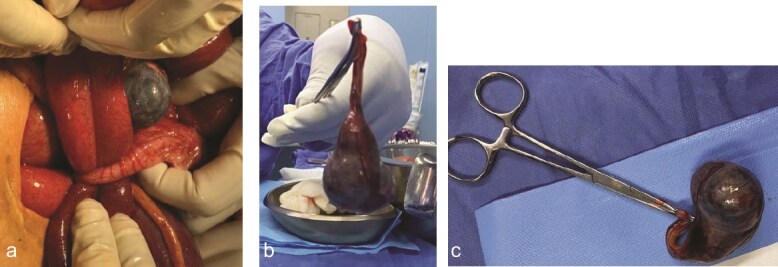
(a) Appendiceal tumor at the tip of the appendix causing strangulation of the distal ileum with discoloration changes. (b, c) An appendix measuring 6 × 1.5 cm with a tumor at the tip measuring 10 × 8 cm.

An exploratory laparotomy identified reactive fluid and an appendiceal tumor at the tip of the appendix causing strangulation of the distal ileum with discoloration changes that reversed the ischemia upon reduction. A Pouchet-type appendectomy was performed, removing an appendix measuring 6 × 1.5 cm with a tumor at the tip measuring 10 × 8 cm (Fig. 2a–c).

**Figure 3:**
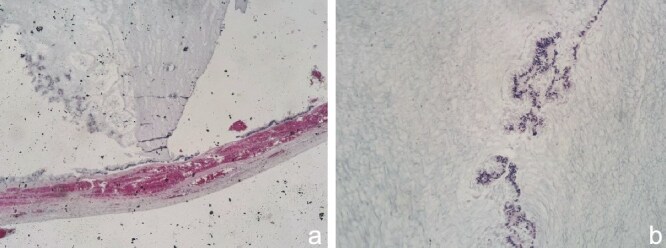
(a) This image shows a longitudinal section of the appendiceal wall. The mucosa is characterized by distension due to the accumulation of mucinous material. The wall appears relatively intact without significant disruption. The mucinous material is stained with hematoxylin and eosin (H&E), indicating the presence of mucin. This is typical of mucoceles, where mucin displaces the normal appendiceal tissue, leading to distension of the organ. (b) This image provides a closer view of the mucosal layer of the appendix, again showing mucinous material. The absence of cellular atypia and the lack of invasive growth into surrounding tissues suggest a benign mucinous neoplasm, possibly a mucocele rather than a mucinous carcinoma. The blue staining likely represents mucin, highlighted with a special stain such as Alcian blue, which is used to identify acid mucopolysaccharides and glycosaminoglycans, typical components of mucin.

Histopathological analysis of the resected appendix revealed the presence of an appendiceal mucocele. The appendiceal mucosa showed increased mucus production with dilation of the appendiceal lumen, characterized by the accumulation of mucin without evidence of cellular atypia (Fig. 3a and b).

The patient experienced a notably favorable recovery immediately postoperatively. Clinical monitoring and laboratory parameters remained within normal limits, allowing for the initiation of oral intake 24 hours after the surgical intervention. No signs of infection or adverse drug reactions were observed. Pain management with nonsteroidal anti-inflammatory drugs was effective, and intestinal function recovered uneventfully. Consequently, the patient met discharge criteria and was successfully discharged on the second postoperative day, with follow-up outpatient appointments for monitoring and scar management.

## Discussion

Intestinal obstruction caused by an appendiceal tumor, as presented in this case, is a rare phenomenon with limited reports in the medical literature. Up to 2018, only 16 cases of intestinal obstructions attributable to mucoceles and other appendiceal tumors, including mucinous cystadenomas [4] and low-grade mucinous neoplasms [5], had been documented. These tumors tend to present at the tip or base of the appendix [6], sometimes complicated by appendiceal intussusception, as observed in seven previously reported cases [7].

Compared to our case, which also involves a mucocele at the tip of the appendix causing strangulation of the distal ileum, the clinical presentation is consistently nonspecific, complicating preoperative diagnosis. Most cases in the literature, similar to our report, do not achieve a definitive diagnosis of mechanical obstruction caused by an appendiceal mucocele until surgical exploration, despite the use of advanced imaging techniques such as CT [1].

Regarding surgical management, there is no clear consensus, reflecting the variety in presentation and severity of cases. In a 2018 review of seven cases studied, two were managed with simple appendectomy while five required more extensive ileocecal resection [5]. This spectrum of surgical interventions suggests that the decision should be based on specific intraoperative findings of the tumor, its extent, and the presence of complications such as local or diffuse mucus collection in the peritoneum. In our case, we opted for an exploratory laparotomy due to the patient's hemodynamic instability, which did not respond to fluid resuscitation and progressively worsened. This decision allowed for a more thorough assessment of the abdominal contents and ensured timely surgical management. Given the clinical presentation and the need for prompt intervention, laparotomy allowed for a more thorough assessment of the abdominal contents and ensured timely surgical management. A Pouchet-type appendectomy was performed based on the mucocele's size and location, achieving favorable postoperative outcomes without complications [8].

This approach aligns with previous studies suggesting that mucoceles >2 cm should be removed due to a higher risk of malignancy. Furthermore, the presence of symptoms and abdominal mass palpable during examination indicate the need for aggressive surgical intervention [8]. Therefore, although simple appendectomy may be sufficient in benign cases, cecal resection or right ileocolectomy might be necessary in more complex situations [3, 5].

In summary, our case contributes to the existing literature by demonstrating that, although the incidence of appendiceal mucoceles causing intestinal obstruction is low, it should be considered in the differential diagnosis of unusual intestinal obstructions. It also emphasizes the importance of tailoring surgical management based on intraoperative findings and the patient's condition [2, 6, 7].
